# Uptake of NHS health check: issues in monitoring

**DOI:** 10.1017/S1463423618000592

**Published:** 2018-08-20

**Authors:** Victoria A. Riley, Christopher Gidlow, Naomi J. Ellis

**Affiliations:** 1 Research Associate and Part-Time PhD Student, School of Life Sciences and Education, Staffordshire University, Stoke-on-Trent, Staffordshire, UK; 2 Associate Professor, School of Life Sciences and Education, Staffordshire University, Stoke-on-Trent, Staffordshire, UK; 3 Senior Lecturer, School of Life Sciences and Education, Staffordshire University, Stoke-on-Trent, Staffordshire, UK

**Keywords:** data quality, NHS health check, uptake

## Abstract

Within the NHS health check (NHSHC) programme, there is evidence of marked inconsistencies and challenges in practice-level self-reporting of uptake. Consequently, we explored the perceptions of those involved in commissioning of NHSHC to better understand the implications for local and national monitoring and evaluation of programme uptake. Semi-structured, one-to-one, telephone interviews (*n*=15) were conducted with NHSHC commissioners and leads, and were analysed using inductive thematic analysis. NHSHC data were often collected from practices using online extraction systems but many still relied on self-reported data. Performance targets and indicators used to monitor and feedback to general practices varied between localities. Participants reported a number of issues when collecting and reporting data for NHSHC, namely because of opportunistic checks. Owing to the perceived inaccuracies in reporting, there was concern about the credibility and relevance of national uptake figures. The general practice extraction service will be important to fully understand uptake of NHSHC.

NHS health check (NHSHC) was implemented as a national cardiovascular disease (CVD) prevention programme in April in 2009 (Department of Health, 2008). The original programme remit was to identify and manage CVD risk in adults aged 40–74 years. All eligible adults should be invited for an NHSHC, in which CVD risk is assessed based on measurements including blood pressure, cholesterol, and other patient information (eg, age, gender, family history, smoking status), is discussed, and used as a basis for subsequent intervention, such as lifestyle advice, GP referral, or signposting to other services.

Uptake of prevention programmes, such as NHSHC, is crucial to show cost-effectiveness for reducing population mortality and morbidity rates. The economic modelling for NHSHC was based on uptake of 75% (Department of Health, 2008), yet five-year cumulative data indicate that uptake of NHSHC is considerably lower (48.5%, 2013–2018; NHS Health Check, 2018). Although uptake has improved as the programme has become more established, it remains an area for attention (Robson *et al*., 2016).

The NHSHC programme standards state ‘timely, good quality data is crucial to establishing robust systems to assess quality and will aid reporting’ (Public Health England, 2014: 11). Unless there are good quality data at a local level, monitoring and evaluation of such preventive health programmes is undermined. Research into UK primary care data quality found that clinical coding systems promoted diversity rather than consistency (Tai *et al*., 2007), and diseases such as coronary heart disease (Bhattarai *et al*., 2012) and stroke (Gulliford *et al*., 2009) showed substantial variation in diagnostic coding, including consultations and referrals. More recently, a review of the NHSHC programme in Croydon identified a need for more efficient data recording and reporting to improve service quality (Brutus, 2013). Our research, which explored uptake and implementation of NHSHC, found that most general practices were unable to accurately report uptake and corresponding figures reported to the local authority for local and national monitoring were often substantially different (Riley *et al*., 2018). This highlighted an important issue to explore; why practices were unable to report NHSHC data accurately and the implications this may have for national monitoring and evaluation.

This report presents findings from interviews with Public Health and Clinical Commissioning Group (CCG) staff to understand how commissioners collect and report programme data and consider implications for national and local monitoring and evaluation. Scoping interviews to discuss the findings and methods for gathering and reporting NHSHC data were initially conducted with four NHSHC leads who the lead author had been in contact with previously. As a result, additional questions were added to the interview schedule. National NHSHC leads, who worked with the authors on a previous study (Riley *et al*., 2018), were then emailed and asked if they would be interested in participating in an interview. In total, 15 semi-structured, one-to-one telephone interviews were conducted with NHSHC commissioners from across England (Midlands and East of England, *n*=3, North of England, *n*=5, South of England, *n*=5, London, *n*=2). In total, 14 participants were employed in public health roles and one was employed by the local CCG.

For inductive thematic analysis, 11 of the 15 interviews were recorded and transcribed verbatim (Braun and Clarke, 2006). This involved familiarisation of data, generation of preliminary codes, and identification of themes before final refinement. All preliminary codes were developed and reviewed by the lead author (V.A.R.) and verified by (N.J.E.), before agreement of initial themes and their relationships. Themes were discussed between the authors (V.A.R., N.J.E.) before being finalised (Table 1).

**Table 1:** Development of themes

Preliminary codes	Initial theme	Finalised theme
Practices not coding an opportunistic invitation	Issues with opportunistic checks	Opportunistic checks
Practices not understanding concept of opportunistic checks		
Electronic extraction is more accurate	View of self-reporting	Accuracy of coding
Looking to move to electronic extraction to improve data		
Unsure on accuracy of coding as self-reported	View of accuracy of coding	
Thinks practices are very good at recording HCs		
Match number of invites to completions if less	Issues with quarterly reporting	Quarterly reporting
Reporting quarterly raises issues with total invitations to completions		

## Experiences of data reporting for NHSHC

NHSHC data were largely collected from practices using online extraction systems (directly or via a third party company; Table 2). The majority of participants said they fed back performance data to practices, most commonly relating to uptake and number of completed NHSHCs. When asked if/what targets are set for practices, they included the number of patients to be invited and/or completed NHSHC. Only five areas (33%) set targets for practices based on uptake. Payments for delivering NHSHC varied. Most provided payments for completed checks, followed by uptake, quality, coverage, and additionally for each patient’s first invitation (five areas).

**Table 2:** Summary NHS Health Check data collection/reporting by participants

Characteristics	*n*
How were data collected by commissioners (*n*=15)?	
Self-reported	6
Using an electronic extraction system	9
Are individual practices fed back information on their performance (*n*=14)?	
Yes	12
No	2
Do individual practice targets include uptake (*n*=14)?	
Yes	5
No	9

A small number of participants had no issues when collecting NHSHC data from practices, largely ‘because we’re using a third party’ (p. 13) data extraction system. Issues experienced by participants when collecting self-report data were thought to be due to ‘practices not using the correct [NHS Health Check] template…so coding doesn’t happen completely accurately’ (p. 10), although those who ‘developed an admin template so they can actually record the invite’ (p. 11) found that the accuracy of their reporting improved. Problems reported when collecting data included clinical errors, practice consent for data-sharing agreements, ineligible patients receiving a NHSHC (ie, those with diabetes, history of CVD), late data submissions, double-coded NHSHCs (ie, completed check coded by pharmacy and the practice), incomplete NHSHCs, and receiving abnormally large figures (eg, 10-fold differences between consecutive quarters).

## Themes

A master theme specifically related to participant’s views and experiences of collecting NHSHC data included ‘accuracy of coding’, ‘opportunistic checks’ and ‘quarterly reporting’. A second theme, separate to the master theme, is called ‘perception of national data’.

### Opportunistic checks

Participants identified issues around coding and practice understanding regarding opportunistic NHSHCs (ie, when patients already visiting the general practice are offered and then immediately receive a NHSHC): ‘this is one of the questions that is asked of me, “if we do an opportunistic [NHS Health Check], we’ve not actually invited the patient” so they won’t actually put the invite code on’ (p. 5). Participants reported that practices did not consider offering a NHSHC to a patient whilst in the surgery to be a verbal invitation. Subsequently, the patient was not coded as receiving an invitation, which has implications for local and national reporting of uptake.

### Quarterly reporting

The combination of opportunistic NHSHCs and the delay in time between patients receiving an invitation and attending a NHSHC also caused problems for participants when reporting quarterly data: ‘a health check received doesn’t correlate for a health check offered’ (p. 8). There was variation in how this issue was addressed; some reported the data submitted to them, whereas others matched the number of invitations to completions (ie, giving 100% uptake): ‘we’ve got deadlines to meet so… I would have just completed a completer as an inviter’ (p. 14). Although the correction may be considered appropriate for quarterly reporting, it poses clear problems for the overall data by increasing the number of invitations and falsely skewing uptake.

### Accuracy of coding

When asked about the accuracy of coding for NHSHC, a number of participants believed that their practices accurately recorded the NHSHC whereas others were ‘not entirely sure because it’s self-reported’ (p. 8). Electronic data extraction was perceived as ‘far more accurate’ (p. 9) as it is more objective and reduces the risk of human error. For this reason, two participants who currently relied on self-reported data were looking to implement electronic data extraction: ‘ultimately we’d like to move to a system where we are getting the data directly from EMIS, which will save work for practices and will give us greater assurance’ (p. 10).

### Perception of national data

When asked about national NHSHC data, some participants believed there is ‘variation with how people are reporting’ (p. 7), which reduced the perceived credibility of national data: ‘I’m not 100% sure that you’re comparing like with like’ (p. 7). For two commissioners, inconsistencies in reporting opportunistic invitations and relying on self-reported data led them to believe the national data ‘may be skewed either negatively or positively by inconsistent coding’ (p. 8). Others thought it was ‘very limited what they [Public Health England] expect back from us’ (p. 11) and it ‘doesn’t tell you anything about the quality’ (p. 12). These participants believed that more data should be reported, such as ‘patient demographics’ (p. 6), to better understand the national NHSHC population.

The majority of participants thought, ‘an awful lot of emphasis [nationally] is from the uptake percentage’ (p. 15), which some considered ‘a meaningless statistic’ (p. 5). There were calls for more focus on ‘how many are eligible and, of those, how many have had a health check’ (p. 5) in the last five years (known as coverage) instead of uptake (percentage of those invited and received a NHSHC). Participants also identified that success of NHSHC varies when uptake is used as a performance indicator: ‘if the local authority uses an opportunistic only model then their uptake is going to be very high vs someone who uses the call and recall system’ (p. 15). Overall, participants appeared to question the validity of national uptake data as a result of varied delivery models and data extraction methods, and the metrics requested by Public Health England.

## Implications of uptake in the NHSHC programme

Overall there was variation in how uptake data were collected, what (if any) performance indicators were fed back to practices, practice targets, and payments for delivering NHSHC. Findings also showed opportunistic NHSHCs created problems with coding of invitations and completed NHSHCs that affected the accuracy of data reported to local authorities. Most striking was that a number of participants did not think uptake should be used as a performance indicator for NHSHC locally or nationally. The apparent lack of importance attached to uptake may explain why practices struggle to provide accurate uptake data (Riley *et al*., 2018) and perhaps why rates of uptake have plateaued at around 50% nationally (48.5%, 2013–2018 data, NHS Health Check, 2018). If few localities set targets based on uptake, do not feedback practice performance in terms of uptake (compared with national target), nor consider uptake to be important, practices may be less likely to prioritise accurate coding of HC invitations, bookings, cancellations, and completions (Riley *et al*., 2018). Nationally, this means current data reported for NHSHC may not be a true representation of programme performance.

A perceived lack of importance of uptake, as seen in our findings, can be compared with findings reported elsewhere. Research exploring quality of clinical coding found barriers, including limitations of coding systems, the time required to record data during consultations, health professional’s motivation to complete the task, and the level of priority given to coding within the organisation (de Lusignan, 2005). If a health professional’s locality do not prioritise the accuracy of recording invitations in order to quantify uptake, they are less likely to be motivated to accurately record NHSHCs. As Bhattarai and colleagues concluded, a high level of data quality is ‘desirable in order to promote good clinical practice as well as to enhance the utility of coded records for researchers’ (p. 5) (Bhattarai *et al*., 2012).

In contrast to previous NHSHC research that focussed on perceptions of GPs and practice managers (Krska *et al*., 2015; 2016), our data from commissioners and NHSHC leads highlight common concerns about the quality and use of routine NHSHC monitoring data. It is important to recognise that our conclusions are based on a small sample and cannot be assumed representative of all. However, our data do make a case for more robust data gathering to fully understand uptake of NHSHC. It has recently been confirmed that Public Health England will be using the General Practice Extraction Service (GPES) ‘to monitor the programme, and help local commissioners and service providers address variation by locality and across different patient groups’ (NHS Health Check, 2017). The GPES may help commissioners and Public Health England to standardise the way data is extracted for NHSHC, which would help to mitigate some of the issues identified with self-reported data identified in this report. Therefore, it represents an important step in improving national data quality for monitoring and evaluation of NHSHC. Otherwise, data quality will remain an issue for commissioners locally, which will continue to affect the quality of national data.
